# Effects of marine heat waves and cold spells on a polar shallow water ecosystem

**DOI:** 10.1038/s41598-025-05621-w

**Published:** 2025-06-20

**Authors:** Philipp Fischer, Holger Brix, Ingeborg Bussmann, Uta Ködel, Max Schwanitz, Claudia Schütze, Norbert Anselm, Markus Brand, Yvonne Jenniges, Sabine Kasten, Alexandra Kraberg, Miriam Lienkämper, Lisa Spotowitz, Ute Weber, Karen Wiltshire, Peter Dietrich

**Affiliations:** 1 Alfred Wegener Institute Helmholtz Centre for Polar and Marine Research, Centre for Scientific Diving, Am Binnenhafen 1117, 27498 Helgoland, Germany; 2 School of Science, Constructor University Bremen gGmbH, Campus Ring 1, 28759 Bremen, Germany; 3 Institute of Carbon Cycles, Helmholtz-Zentrum hereon GmbH, Max-Planck-Straße 1, 21502 Geesthacht, Germany; 4 Alfred Wegener Institute Helmholtz Centre for Polar and Marine Research, Section Shelf Sea System Ecology, Kurpromenade 212, 27498 Helgoland, Germany; 5 Department Monitoring and Exploration Technologies, Helmholtz-Centre for Environmental Research UFZ, Permoserstr. 15, 04318 Leipzig, Germany; 6 Department Computational Hydrosystems, Helmholtz-Centre for Environmental Research UFZ, Permoserstr. 15, 04318 Leipzig, Germany; 7 Alfred Wegener Institute Helmholtz Centre for Polar and Marine Research, Computer and Data Center, Am Handelshafen 12, 27570 Bremerhaven, Germany; 8 Alfred Wegener Institute Helmholtz Centre for Polar and Marine Research, Section Ecological Chemistry, Am Handelshafen 12, 27570 Bremerhaven, Germany; 9 University of Bremen, Bibliotheksstraße 1, 28359 Bremen, Germany; 10 Alfred Wegener Institute Helmholtz Centre for Polar and Marine Research, Section Marine Geochemistry, Am Handelshafen 12, 27570 Bremerhaven, Germany; 11 Faculty of Geosciences, University of Bremen, Klagenfurter Straße 2-4, 28359 Bremen, Germany; 12 Alfred Wegener Institute Helmholtz Centre for Polar and Marine Research, Section Polar Biological Oceanography, Am Handelshafen 12, 27570 Bremerhaven, Germany; 13 Alfred Wegener Institute Helmholtz Centre for Polar and Marine Research, Section Coastal Ecology, Hafenstraße 43, 25992 List/Sylt, Germany; 14 Environmental and Engineering Geophysics, University of Tübingen, Schnarrenbergstr. 94-96, 72076 Tübingen, Germany

**Keywords:** Phenology, Marine biology, Scientific data

## Abstract

Global warming affects the Earth system in complex ways, often preventing a functional understanding of the underlying processes. Disentangling these processes between abiotic drivers and single species or entire communities is, however, essential for an in-depth understanding of the impacts of climate change on the ecosystem. Using a high-resolution time series on heat waves and cold spells in an Arctic fjord system, we demonstrate that AI-supported digital data processing, which is based on state-of-the-art observatory technology, has the potential to provide new insights into the effects of abiotic factors on biotic communities, which would not be possible with traditional expedition-based sampling methods. Furthermore, our study shows that short-term, event-driven anomalies in key ocean variables not only alter a system’s hydrography but also have the potential to impact the entire community across the trophic chain from benthos and zooplankton to fish. We found a significant positive correlation between hydrographic temperature anomalies and biota abundance, with high biota abundances linked to ‘Atlantic’ phases with frequent heat waves and low biota abundances correlated with ‘Arctic’ phases dominated by cold spells. The study also revealed that hydrographic anomalies can not only influence overall biota abundance in an area but also trigger complex shifts in species composition. This leads to fluctuating interannual abundance peaks in specific biotic groups, such as jellyfish, fish, or chaetognaths, depending on trigger factors that are not yet fully understood.

## Introduction

Establishing numerical relationships regarding the effects of climate change on aquatic ecosystems is a challenge^1^. While methodologies for assessing essential ocean variables (EOVs), including temperature, salinity, and partial pressures of greenhouse gases (e.g., methane and carbon dioxide), have advanced in recent decades, our understanding of the effects of these variables on higher trophic levels remains limited^2,3^.

This is particularly true for coastal ecosystems, which exhibit comparatively high structural and hydrographic complexity^4^. High-frequency in situ observations of the mobile fauna are still limited^5^. In addition, the behavioural and physiological responses of higher-trophic-level organisms, such as fish, to gradual long-term trends in the environment often do not result in spontaneous or obvious changes in overall abundance and behaviour in a given area. Instead, they result in incremental changes, e.g., in the growth or survival rate of a particular species or age class. In addition, such behavioural or physiological responses may exhibit an unknown temporal and spatial shift^6^ making the relationship between changes or fluctuations in the abiotic environment and community responses even more complex. These changes may only become apparent after a certain period, e.g., at the end of the year when the growing season is over, or even later in the lifespan of a species through a change in the year class strength of a particular cohort.

Numerous studies on species and community responses to climate change^7,8^ have emphasized the need for long-term time series and high-frequency sampling^9^ to disentangle interactions between seasonal, interannual or even decadal and random fluctuations in population dynamics from those of long-term environmental trends due to climate change. For example, Hobday^10^ postulated that monitoring the effects of climate change on communities requires at least thirty years of observations to significantly discriminate seasonal or random fluctuations in community structure from long-term changes due to climate change. Recent studies have shown that long-term trends are only a partial reflection of the response of the Earth system to climate change^3,11^. In recent years, hydrographic anomalies, such as heat waves, cold spells, storm surges and droughts, have become more prominent^6,11,12^. Within this context, the question of classifying environmental anomalies as fluctuations, anomalies or extreme events is being increasingly discussed^13^. The IPCC^14^ provides highly valuable considerations for a meaningful classification of semantic separation, focusing on the impact level of an anomaly^13^ and, second, the rareness of an event. However, the authors also stated that the impact level is highly dependent on the context and event type. This is especially true for remote areas, e.g., polar waters, as little is known about the long-term effects of short-term hydrographic anomalies on ecosystems, particularly trophic cascades^3^. Therefore, a more tangible criterion, especially in these areas, might be the frequency of occurrence of anomalies. In disciplines with long-term datasets (> 100 years) and extensive areal and temporal data coverage, such as the atmospheric sciences, extremes are derived from probability distributions of the annual maximum values of the target variables^15^. In remote areas with sparse data availability, a more frequency-based statistical approach is common for separating anomalies from extremes^10^ often based on values below the 10th percentile or above the 90th percentile of a certain period (day, month, or year) against a longer-term average. This approach is especially common for defining marine heat waves^16^. Independent of the semantic classification, the impact of hydrographic anomalies on a marine ecosystem depends on their duration and amplitude compared with the timescales of the ecological response and the sensitivity of different ecosystem components to environmental changes. Unfortunately, most of these considerations of the impacts of climate-induced anomalies on environmental parameters are derived from theoretical models and lack observational evidence^17^.

In this work, high-frequency time series from the Arctic Kongsfjorden system in Svalbard are used to assess the potential effects of short-term regional hydrographic anomalies on the shallow-water coastal community. To avoid the pitfalls of a semantic debate on the definitions of “extreme” and “anomaly”, we have opted to use the term “anomaly” throughout the manuscript. This is in line with the methodology established by Wakelin^16^ for identifying marine heat waves, which we adapted to encompass anomalies in water temperature, salinity, and total and species-specific biotic abundances in a polar fjord ecosystem (see Online Materials and Methods).

The Kongsfjorden ecosystem, located at the boundary between the Atlantic and Polar spheres, is described as a hotspot of climate change in the Arctic^18^. It is characterized as an alternating ecosystem with periodic massive inflows of warmer and more saline water from the North Atlantic Current (NAC) and polar phases when colder and less saline water from the cold East Spitsbergen Current (ESC) dominates the fjord system^19^. These large-scale temporal changes in the fjord’s hydrographic regime (hereafter referred to as “Atlantic phases” or “Arctic phases”) are superimposed by local or regional phenomena, such as wind-driven coastal upwelling or downwelling^20^ resulting in distinct hydrographic events.

**Fig. 1:**
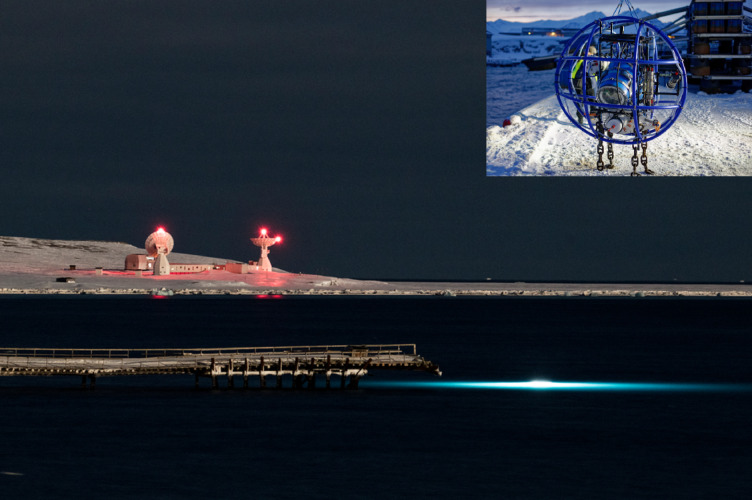
AWIPEV shallow-water cabled underwater observatory (here, artificially illuminated during a maintenance period in the polar winter of 2021) in front of the “Old Pier” near Ny-Ålesund on the southern coast of the Kongsfjorden ecosystem at 78°54.200 N and 11°54.00 E. The basement of the system is located at a water depth of 12 m and consists of the water inlet of a land-based FerryBox system and a vertical profiling system in which the main essential ocean variables (EOVs), water temperature, salinity, oxygen saturation, chlorophyll A and turbidity, are measured between a water depth of 11 and 0 m year-round together with high-resolution stereoscopic images recorded every 30 min. The vertical profiling unit is shown at the inlet. Photo: Photographed 2022 by Gregory Tran and Max Schwanitz (inset) on behalf of Alfred-Wegener-Institute.

Since 2012, the Alfred Wegener Institute (AWI) has been operating a shallow water cabled underwater observatory on the southern coast of Kongsfjorden at 78°54.200 N and 11°54.00 E (Fig. 1). In close collaboration with the Helmholtz-Zentrum Hereon (HEREON) and the Helmholtz Centre for Environmental Research (UFZ), as well as within the framework of the observational infrastructures COSYNA^21^ and MOSES^22 ^the main EOVs (water temperature, salinity, oxygen saturation, chlorophyll A and turbidity) are sampled all year at a water depth of 11 m,  with a frequency of 1 Hz. In addition, a vertical sensor profiler samples the same EOVs at the same frequency once a day at noon, moving from the maximum depth to the surface (for more details on the underwater sensor system, see^23^).

Furthermore, a stereoscopic observatory for macrofauna and fish is integrated into the system, and stereoscopic image pairs are taken every 30 min. These image pairs are semiautomatically analysed with AI support for total and group-specific biotic abundance (fish, jellyfish, appendicularia, pelagic crustacea, benthic crustacea, chaetognatha, pteropoda, and others). The grouping of these eight biota categories was based on the observation that the members of each group belonged to the same phyletic lineage, consistently appeared in higher abundances at a specific time of year over the entire observation period and, during this time, formed a dominant part of the community in terms of total biota abundance. Similar to the calculation of abiotic short-term anomalies, we also calculated anomalies for these biotic groups as well as for the individual species via a modified Hobday approach (see Online Materials and Methods).

We used these datasets to test whether short-term anomalies in the essential ocean variables water temperature and salinity significantly affect the shallow water community. We tested for a significant functional relationship between the frequency and intensity of water temperature and salinity anomalies in the area and the associated community in terms of biota abundance. We then tested whether anomalies in water temperature and salinity affect individual biotic groups or the entire shallow-water community.

## Results

**Fig. 2:**
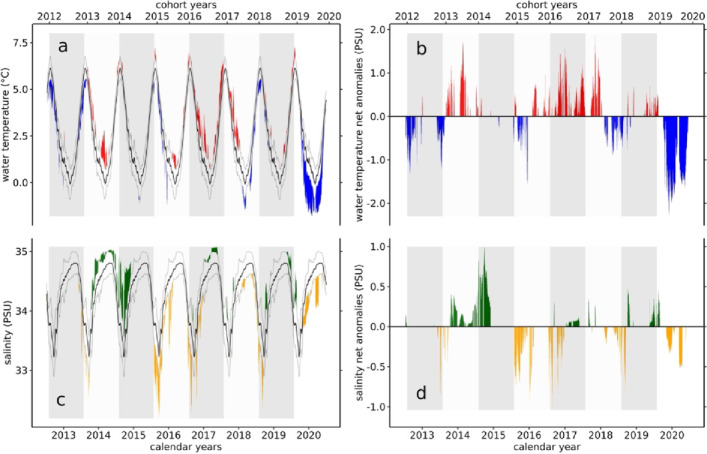
(Panels **a** and **b**) Visualization of the water temperature and salinity time series from 2012–2020, including the anomalous phases calculated after Hobday^10^. The black lines indicate the long-term average over the entire sampling period. The grey lines show the upper and lower 90% confidence limits of the long-term average. In Panel a, the red areas represent heat waves, and the blue areas represent cold spells. In Panel **c**, the green areas represent phases of anomalously high salinity, and the yellow areas represent phases of anomalously low salinity. In Panels **b** and **d**, the net anomalous values are shown with the same colour codes as those in Panels **a** and **c**. The grey and white boxes represent the cohort years, each lasting from 01 August of a year to 31 July of the following year (see Online Materials and Methods for details).

The daily mean water temperatures and salinities from 2012 to 2020 at the study site within the Kongsfjorden system (see Online Material and Methods) are shown in Fig. 2.

Additionally, the 90% confidence intervals (CIs) and the absolute and net anomalous values of water temperature (Fig. 2, Panels a and b) and salinity (Panels c and d), calculated after Hobday^10^ are shown. Mann–Kendall time series trend analysis^24^ revealed that, contrary to the overall increase in absolute water temperature in the area shown in previous studies^25 ^no trends in the frequency (temperature τ = 0.113, *p* = 0.16; salinity τ = -0.008, *p* = 0.92) or intensity of water temperature or salinity anomalies (heat waves, τ = 0.0289, *p* = 0.73; cold spells, τ = -0.0794, *p* = 0.36; high-salinity phases, τ = -0.147, *p* = 0.09; low-salinity phases, τ = -0.151, *p* = 0.08) were observed over the sampling period. However, Fig. 2 clearly shows distinct seasonal and longer-term temperature and salinity patterns. On a seasonal scale, high temperatures and low salinities were observed in summer, whereas low temperatures and high salinities occurred in winter. Additionally, longer-lasting patterns in water temperature and salinity also occurred. The 2013 and 2014 cohort years as well as the 2016 and 2017 cohort years were dominated by heat waves, whereas the years 2012, 2015, 2018 and 2019 were characterized by cold spells.

**Fig. 3:**
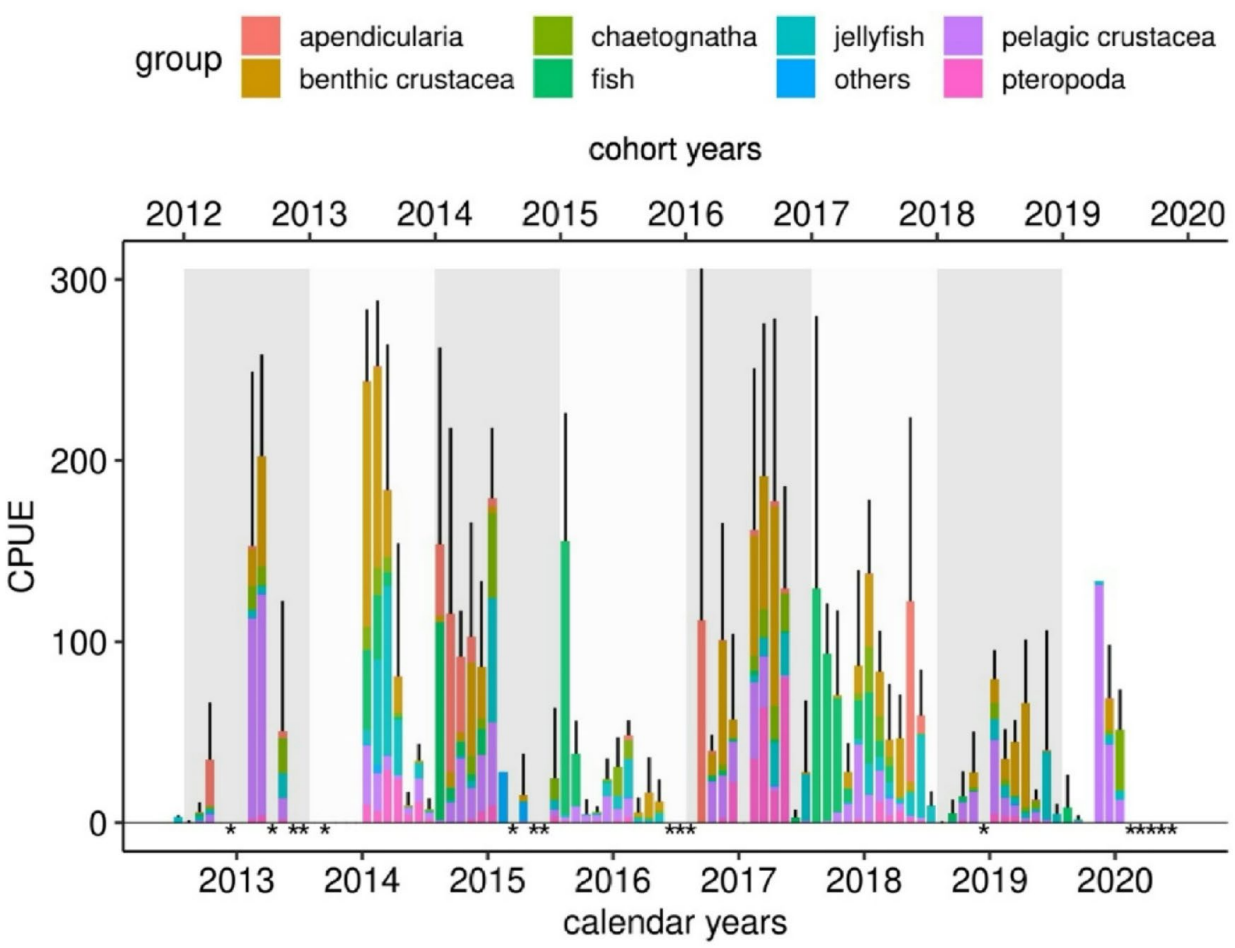
Monthly mean abundance (total bar height) and standard deviation (black error bars) of organisms calculated from the total number of organisms counted on the stereoscopic images per week (catch-per-unit-effort/CPUE) from July 2012 to January 2020. The proportions of the different biota groups per month are represented by the different colours of the bars. * indicates months when no data were available due to technical failure of the observatory. The grey and white boxes represent the cohort years (labelled above), each lasting from 01 August of a year to 31 July of the following year.

The continuous stereo-optical recording of the shallow-water zone of Kongsfjorden allowed good estimation of the biological activity in the area. Using the total and group-specific abundance values per week (catch-per-unit-effort/CPUE) as replicates, the monthly mean CPUE (+/- SD) was calculated from July 2012 to December 2019. Figure 3 shows the total abundance of organisms per month divided into the different biota groups. As the biological year in the Arctic realm lasts from summer to summer with the highest abundance data in the winter months^26^ we also superimposed the 2012–2019 cohort years as biological units in Fig. 3, with each biological cohort year lasting from 01 August of the year to 31 July of the following year (see also Online Materials and Methods).

The results demonstrate distinct interannual variations in overall organism abundance. The 2012–2014, 2016 and 2017 cohort years were characterized by higher overall abundances, whereas 2015, 2018 and 2019 presented lower overall abundances. When the total abundances of the different groups were separated, seven highly abundant groups emerged: appendicularia, chaetognatha, decapoda, pelagic crustacea, pisces, pteropoda and scyphozoa.

**Fig. 4:**
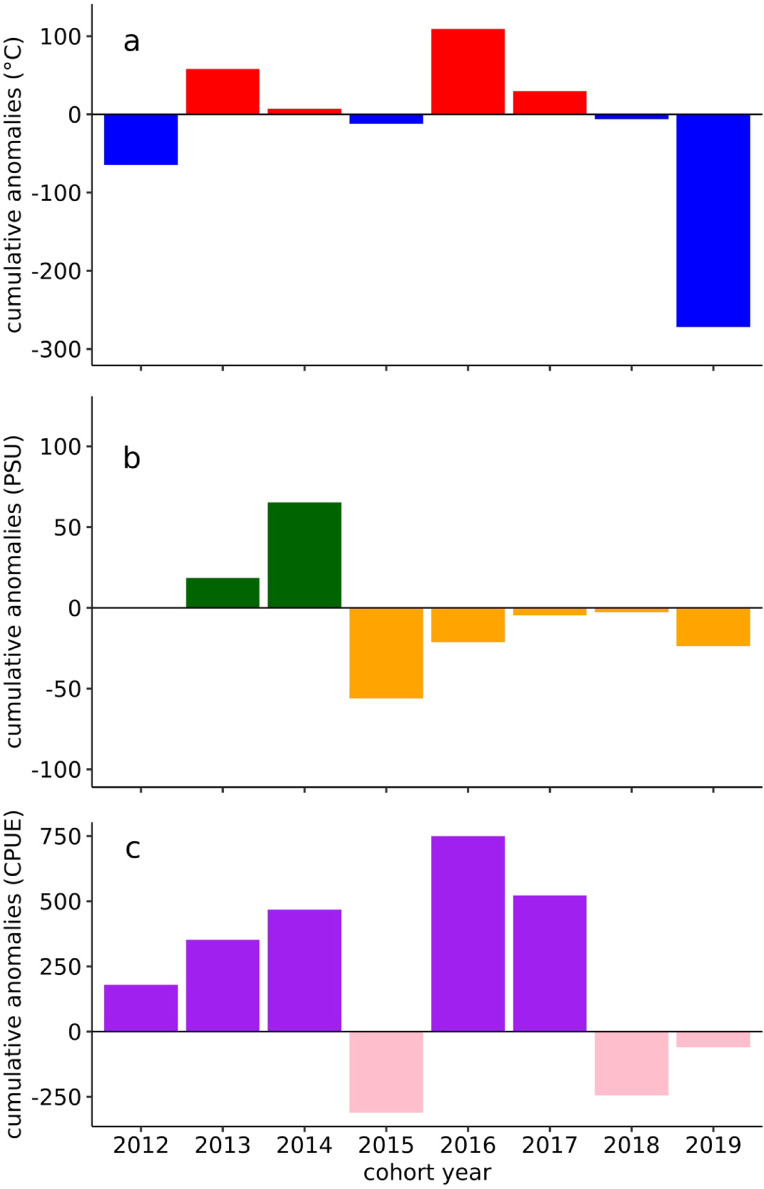
Cumulative anomalous values per cohort year for temperature (°C; **a**), salinity (PSU; **b**) and biotic abundance (CPUE; **c**). Two different colours are used in each panel to better illustrate positive and negative anomalies in the plots. .

Higher overall abundances were generally observed in the fall and winter months, whereas lower abundances were observed in spring and early summer. Figure 4 shows the cumulative sum of anomalous values in the cohort years for temperature (a), salinity (b), and the combined biotic abundances (c). In all three panels, positive values indicate an exceedance of the measured values (water temperature, salinity, or biota abundance) above the upper 90% percentile value calculated from the long-term average from 2012 to 2020, whereas negative values indicate a decrease below the lower 90% percentile value of the long-term average (for details on the calculation of positive and negative anomalies in water temperature, salinity, and biota abundance in the areas, see Materials and Methods as well as^10^).

Considering the abiotic variables, temperature and salinity, different scenarios can be distinguished: (a) cohort years dominated by cold spells and a low- or neutral-salinity climate (2012, 2015, 2018 and 2019), (b) cohort years dominated by heat waves and a high-salinity climate (2013 and 2014) and (c) cohort years dominated by a low-salinity climate but with an overall dominance of heat waves (2016 and 2017). In summary, half of the cohort years from 2012 to 2019 (2013, 2014, 2016 and 2017) were characterized by heat waves, whereas the others (2012, 2015, 2018 and 2019) were dominated by cold spells (Fig. 4a). The cohort year with the most anomalous days was 2019, with a cumulative sum of 271 anomalous cold days. Another notable phase was from 2014 to 2015. While changes in the salinity regime in all other years changed rather moderately from one year to the other, from 2014 to 2015, an abrupt change in the salinity regime was observed from a phase dominated by an increasing number of high-salinity anomalies from 2012 to 2014 to a phase dominated by low-salinity anomalies (Fig. 4b). This change in water masses from a high- to a low-salinity regime occurred in the summer of 2014.

Figure 4c illustrates the occurrence of abundance anomalies in the community from 2012 to 2020. In seven of the eight cohort years (all but 2012), the observed dominance of positive or negative anomalies in overall biotic abundance coincided with a dominance of positive or negative anomalies in water temperature. In the 2012 cohort, a decoupling of temperature and abundance anomalies was observed, with an overall dominance of cold waves and a dominance of positive anomalies in biotic abundances.

**Fig. 5:**
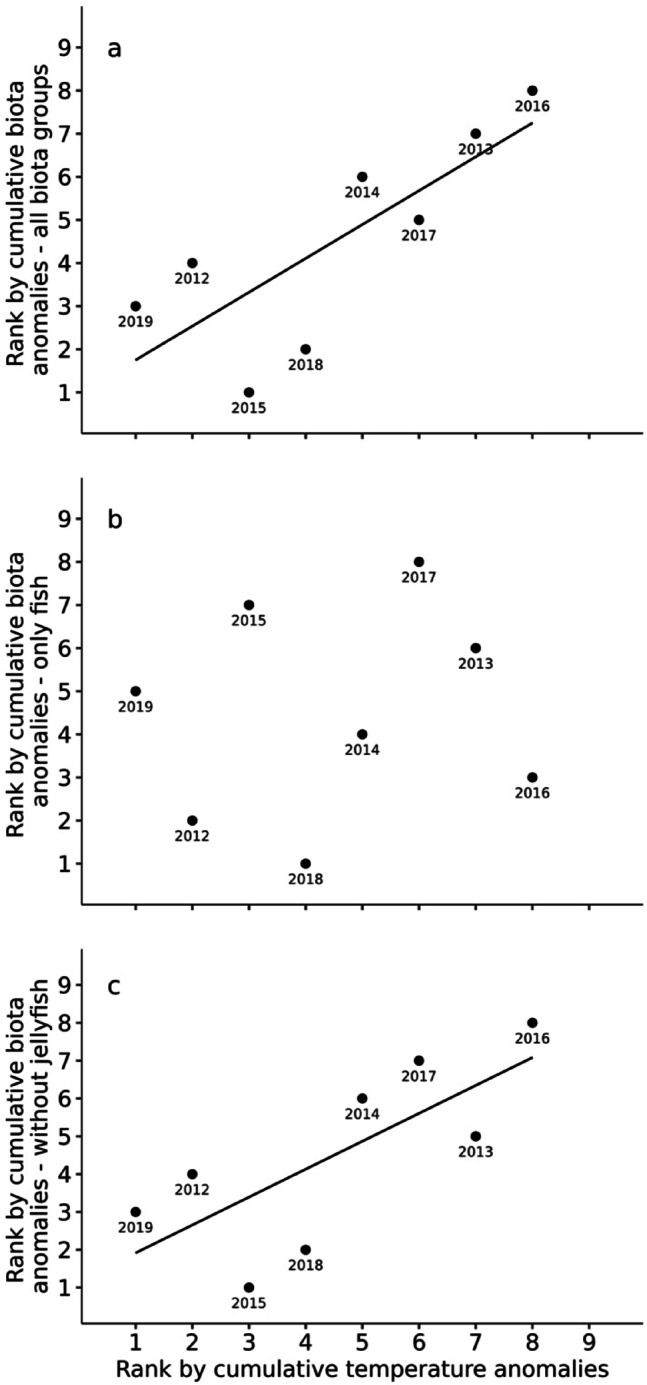
Rank correlation between the intensity of temperature anomalies and the occurrence of abundance anomalies in the different cohort years from 2012–2019 for total biotic abundance (**a**), fish abundance (**b**), and biotic abundance without jellyfish (**c**). Ranks for temperature anomalies were calculated on the basis of the cumulative sum of the duration (days) and intensity (°C) of temperature anomalies for each year (day degrees; see Online Materials and Methods for details).

Statistical analysis confirmed a significant positive correlation between temperature anomalies and total biotic anomalies (Fig. 5). Regression analysis of the ranks of temperature (most anomalous cold spell = lowest rank; most anomalous heatwave = highest rank) and biotic anomalies revealed a highly significant positive correlation between the occurrence of temperature anomalies and biotic anomalies (Fig. 5a, F(df 1,6) = 9.68, p < 0.01), with an adjusted r^2^ value of 0.55. In contrast, salinity was not significantly correlated with biotic anomalies (p = 0.45). When the biotic groups were analysed separately, 7 of the 8 biotic groups did not show any correlation between temperature and abundance anomalies (Table 1), as exemplified by the ‘fish’ group (Fig. 5b). Only jellyfish showed a significant positive correlation between temperature and abundance anomalies (Table 1), despite slightly different rankings of the abundance values across the years. To elucidate the effect of jellyfish abundance on the correlation between temperature and biota anomalies, regression analysis was also performed without the “jellyfish” group (Fig. 5c). This analysis revealed that the strong positive correlation between temperature anomalies and biotic anomalies persisted even without jellyfish (Fig. 5c, F(df 1,6) = 7.18, *p* < 0.03), with an adjusted r^2^ value of 0.48. Although the p and r^2^ values of the analysis without jellyfish were slightly lower than the values for all biota groups, the analysis suggested that jellyfish were not the driving factor for the observed correlation between temperature anomalies and the occurrence of total biota anomalies.

**Table 1:** Regression statistics of the rank correlation between the intensity of temperature anomalies and the occurrence of biotic anomalies in the different cohort years from 2012–2019 separated by biotic group. Ranks for temperature anomalies were calculated on the basis of the cumulative sum of the duration (days) and intensity (°C) of temperature anomalies for each year (day degrees; see online materials and methods for details). Ranks for the biotic anomalies of the different groups were calculated on the basis of the cumulative sum of the biotic anomalies in the respective biotic group (sum of the CPUE above or below the 90% confidence limits of the expected CPUE in the group) (see the online materials and methods for details).

Biotic group	Total number of organisms analysed	F(1,6)	adj.*r*	slope	t value	*p* value of slope
All biotic groups integrated	18 624	11.41	0.59	0.81	3.38	0.01**
Appendicularia	1 866	0.64	-0.06	0.31	0.80	0.46
Benthic crustacea	4 616	2.57	0.18	0.54	1.60	0.16
Chaetognatha	1 286	0.05	-0.17	0.10	0.23	0.82
Fish	3 321	0.06	-0.16	0.09	0.23	0.82
Jellyfish	2247	11.41	0.60	0.81	3.38	0.01**
Pelagic crustacea	3525	0.003	-0.17	-0.02	-0.06	0.96
Pteropoda	1595	2.90	0.21	0.57	1.71	0.14
Others	168	0.29	-0.11	0.21	0.54	0.61

## Discussion

This study provides a glance at the potential of disentangling complex relationships between climate change-induced abiotic trends and events with the associated shallow-water community when data of sufficient temporal resolution are available. We used state-of-the-art underwater observatory technology and quality-controlled temperature and salinity data combined with AI-supported stereoscopic imaging technology with a temporal resolution of 30 min. With these data, we were able to prove significant correlations between biota abundances in the Kongsfjorden ecosystem and the frequency of water temperature anomalies, which were calculated according to the definition of heat waves by Hobday^10^. This finding, facilitated by the use of AI technology, suggests that digitalization can enhance marine sciences, especially in areas where data acquisition is difficult due to remoteness, such as polar areas. Additionally, the study shows that state-of-the-art digital observatory technology opens a window for a review of the often-cited need for “at least 30 years” of sampling^10^ when addressing climate change topics. In a previous study^25^ using the same dataset as in the present study, we reported a significant increase in sea water temperature of approximately 0.22 °C y^− 1^ over a period of only eight years (2012–2018), a disconcertingly high temperature increase. This finding was confirmed by independent high-frequency mooring-based measurements showing an increase of 0.14 °C year^− 1^ in the deeper water layers of the Kongsfjorden ecosystem^27^. However, in the same study^25 ^we also showed that this increase would not have been detectable if sampling was performed only once a week, a sampling frequency that is still high compared with normal sampling frequencies based on non-permanent sampling, e.g., expedition-based sampling. This is because a lower sampling frequency reduces the statistical power of an analysis and thus reduces the probability of detecting significant changes in the target variables over time^28^. This shows that consistent digitalization in marine science, including the use of new digital approaches and solutions for continuous and rapid-response sensor technology, is an evolving key technology in ocean science that is necessary to maintain our knowledge of the effects of climate change at a level that allows us to keep pace with its accelerating impact on the Earth’s ecosystem.

Digitalization has the potential not only to increase the statistical power of time series analysis of key ocean variables but also to enhance the analysis of ecological data, with applications in species interactions and environmental variables^29^. This approach is most advantageous, as quantitative analyses of the relationships between abiotic anomalies and the biotic environment are highly complex and often complicated by an unknown temporal offset. For the present study, we used a dataset from the Kongsfjorden ecosystem for an in-depth analysis of such complex abiotic–biotic couplings. This study revealed that temperature anomalies in marine environments can have a sustained and significant impact on the community. In our study, however, this relationship emerged only when the organisms were summarized across biota groups at the community level. At the group level, a significant correlation was found in only one out of eight biotic groups (jellyfish), and no correlations were found at the species level. This finding indicates that the discriminatory power of the applied analysis increased with the integration level of the data on the temporal scale (abiotic and biotic variables were accumulated over one cohort year) and on the numerical scale (abundances were pooled across all species). The emergence of a notable positive correlation solely at a high temporal integration level implies a temporal shift in the community’s response within the time scale of a “cohort year”. Furthermore, the fact that the correlation appeared only when abundances were aggregated across all groups at the community level suggests a response at the community level rather than at the species or group level.

In an extensive review, Del Monte-Luna et al.^30^ discussed such responses at the community level, referring to them as community carrying capacity (CC). The CC hypothesis, originally proposed by Verhults^31^ postulates a density-dependent community structure in which individual species within the community undergo sequential structural changes influenced by both interactions among species and among species and the environment. In our study, this would mean that in the case of positive temperature anomalies (higher than the 90% confidence interval of the long-term temperature average), the carrying capacity of the system increases, and an abnormally large amount of biomass is possible in the area. In contrast, in the case of negative temperature anomalies (lower than the 90% confidence interval of the long-term temperature average), the carrying capacity of the system decreases to an anomalously small biomass. The hypothesis of a community carrying capacity-regulated system would imply that during such anomalies, the increase in total biota abundance is not fixed with respect to a specific community composition. Instead, different species or groups can show different biomass changes depending on the environmental conditions at that particular moment.

The groups or even individual species that can build more biomass or remain behind at a particular time may depend not only on the actual community composition and their mutual interactions^32^ but also on external factors, such as a possible current-related inflow of larvae of a particular species. However, as soon as the community carrying capacity of the system is reached, the community runs into a (numerical) restriction of further increases in biomass^30^. A limiting process, most likely based on competition for resources, then intervenes and changes the community composition towards a particular species or group with the highest competitive potential, leading to anomalous abundances of a certain group or species. The successful group(s) or species, however, can differ every year (or temporal phase) depending on the respective environmental conditions at a certain time, thus resulting in greater overall variability in the community over time.

Owing to this increasing complexity in community carrying capacity calculations, Dhondt^33^ even recommended avoiding the term “community carrying capacity” at all, as its interpretation becomes too complex and vague and sometimes even implies unresolved contradictions. Our study reveals such an unresolved contradiction. We found (1) a positive correlation between temperature anomalies and total abundance anomalies and (2) a positive correlation between temperature anomalies and jellyfish anomalies, whereas none of the other biota groups showed such a correlation. Thus, one might expect that the jellyfish group drives the correlation between temperature anomalies and total abundance anomalies. However, when the jellyfish group was excluded from the analysis, the positive correlation persisted. This finding indicates that jellyfish do not drive biotic anomalies, which contradicts the primary assumption. We are not yet able to resolve this apparent contradiction here. One hypothesis is that jellyfish do not strongly rely on shallow water community resources and therefore do not significantly contribute to the carrying capacity of the shallow water system in the Kongsfjorden ecosystem. However, this may also hold true for the appendicularia group.

Nevertheless, as environmental–community interactions are becoming even more complex when climate change is considered, addressing factors influencing the carrying capacity of ecosystems together with an adequate sampling scheme^25^ has evolved into one of the hot topics of modern ecology^34,35^ with often not yet fully understood ingredients^30^.

Although our study revealed that, compared with water temperature, salinity anomalies seem to have only minor effects at the community level, the results indicated that salinity anomalies have the potential to amplify or attenuate temperature effects. In 2016 and 2017, numerous low-salinity anomalies were observed in the area (Fig. 4b). Svenson^36^ and Cottier^20^ explained this phenomenon as glacier meltwater runoff with a high particulate content, leading to additional surface water warming by insolation, amplifying the already elevated and temperature driven biotic production in shallow-water systems. Our assumption that salinity and other abiotic variables can act indirectly as cofactors amplifying or muting primary (here, temperature) effects on the community is supported by the literature, which indicates that glacial meltwater predominantly stimulates primary and secondary production in Arctic fjords, either through increased nutrient input or stronger stratification^37^.

Another interesting observation supporting the community carrying capacity hypothesis is provided by Konik^38^ for the same area. Their study on interannual chlorophyll-a (chl-a) concentration patterns (as determined by remote sensing) revealed a positive correlation with our overall biotic abundance data from 2012 to 2020. In particular, the positive anomalies in chl-a concentrations in 2016 and 2017 detected by Konik^38^ coincide well with our positive overall biotic anomalies in these years. As the overall chl-a concentration in a marine area is a proxy for potential overall primary production^39^ our observed high overall biotic abundances in these years may be the result of the relatively high carrying capacity of the area due to increased primary production.

To our knowledge, this study is one of only a few in situ studies that show that anomalous or extreme events, particularly those in opposing directions (cold spells versus heat waves), which are partly amplified or muted by additional cofactors (here, salinity), can have a significantly longer lasting impact on marine communities across the entire trophic chain. Furthermore, it provides an example of an actual heavily discussed topic in community ecology: the entanglement and numerical discrimination of abiotic and biotic factors regulating community structure by determining the carrying capacity of an ecosystem. As environmental–community interactions are becoming even more complex when considering accelerating climate change processes, especially in the Arctic, understanding these processes is most important for improving our predictive power with respect to environmental changes not only at the abiotic level (temperature) but also for the biotic cascade across trophic levels^25,30,34,35^.

As the Arctic ecosystem is often used as a fast-track model for studying the effects of climate change on the Earth system, the results presented here may have wider implications for understanding ecosystem responses to climate change in other regions. As requested by Del Monte-Luna et al.^30^,our study contributes to a better understanding of the relationship between environmental stressors and biological responses across cohort years and contributes to a functional understanding of the effects of climate change on ecosystem resilience. However, a comprehensive and numerical understanding of these complex effects at appropriate temporal and spatial scales is still in its infancy, and the potential of analysing these data is far from exhausted.

Notably, it would have been impossible to detect the relationships between abiotic anomalies and the shallow-water community with data from expedition-based campaigns. With the ongoing process of digitization in marine environmental science, it is becoming possible to generate such high-resolution datasets over several years. This, coupled with the integration of modern AI-based technologies, particularly automated image processing, allows even complex datasets to be comprehensively analysed within an acceptable time. These technologies need to be further developed to maintain our knowledge of the effects of climate change at a level that allows us to keep pace with its accelerating impact on the Earth’s ecosystem.

With respect to the presented data, multiple possibilities exist for further detailed analysis. In this study, we focused on the relationship between long-term temperature anomalies and the occurrence of anomalies in the shallow-water community. For this, we rank-transformed the data across all groups and removed much of the internal variability. Even though this seems to be a crude procedure in community data analysis, as variability is an important part of biological reality, it allows the shaping of complex data with a large internal unknown variability in such a way that long-term patterns may become visible. As the variability in temporal or spatial data at the community level is often very high, making the analysis increasingly complex^30^ we believe that such simplification procedures are acceptable as a first step towards an in-depth multivariate analysis.

In addition to the community-level analysis, our dataset provides detailed information on the body length or size of all the organisms. The morphometric values for each organism (e.g., the standard length of a fish) were determined using AI-based image analysis software and stereogrammetric algorithms^40–42^. These measurements enable the calculation of size‒frequency distributions and, in the case of local communities, average growth rates for each species, supporting an in-depth analysis of species‒specific seasonal changes in size‒frequency distributions within and between years. However, as discussed earlier, while this analysis would be highly valuable, it is beyond the scope of this manuscript and will need to be addressed in a follow-up study.

### Summary

This study demonstrated that large-scale hydrographic anomalies caused by massive water mass influxes from either the Atlantic or Arctic realm significantly influence not only the hydrography of a system^20^ but also the entire ecosystem across the trophic chain. A strong positive relationship was observed between anomalously high biota abundances and “Atlantic” phases, which were characterized by frequent heat waves, A similar relationship was identified between anomalously low biota abundances and “Arctic” phases dominated by cold spells. However, exceptional years where these patterns appear to be influenced by unknown additional factors were identified. In addition to biotic interactions, this work highlights the potential of AI-assisted underwater observatory technology. The combination of high-frequency, year-round sampling infrastructure with datasets on water temperature, salinity, macrofauna, and fish communities revealed that short-term temperature and salinity anomalies exert measurable and significant impacts on biotic communities. Nevertheless, this study also underscores the challenges of analysing high-resolution ecological datasets that integrate abiotic and biotic time series. AI technologies, particularly for images but also for data analysis, can greatly enhance the evaluation of such complex datasets. These advances in analytical methods, together with modern sensor systems, will help ensure that even complex species‒environment relationships can be better analysed and understood in the future.

## Materials and methods

All data used in this manuscript were from the COSYNA-MOSES-AWIPEV cabled Underwater Observatory (Fig. 1, AWIPEV UWO)^21–23^. The observatory has been located in the Svalbard Archipelago in Kongsfjorden at 78°55.200 N 11°54.00 W, at 11 m water depth (± 0.7 m SD tidal amplitude) on the west coast of Spitsbergen since July 2012^26^. The site is sheltered in the inner part of Kongsfjord, with a mean tidal current of 0.1 m s^− 1^,^23^. The fjord system is located at the boundary between the Atlantic and Polar regimes and is described as a hotspot of climate change in the Arctic^18^. It is characterized by an alternating ecosystem with periodic massive inflows of warmer and more saline water from the North Atlantic Current (NAC) and polar phases when colder and less saline water from the cold East Spitsbergen Current (ESC) dominates the fjord system^19^. These large-scale temporal changes in the fjord’s hydrographic regime are superimposed by local or regional phenomena such as wind-driven coastal upwelling or downwelling^20^resulting in distinct hydrographic events.

EOVs (temperature, salinity, oxygen saturation, chl-a, turbidity, current and light (PAR)) are recorded at the AWIPEV-UWO at a frequency of 1 Hz throughout the year and published as annual quality-controlled datasets with a resolution of one hour in PANGAEA^43–51^. On the basis of the temperature and salinity data, heat waves and cold spells from 2012 to 2020 were calculated according to the recommendations and algorithms of Hobday^10^. With this method, the mean water temperature and mean salinity were measured each day from 2012 to 2020. In addition, a long-term reference value with upper and lower 90% percentile ranges for temperature and salinity for each day of the year was calculated from the daily temperatures and salinities from 2012 to 2020. In accordance with Hobday^10 ^phases were defined as heat waves when the measured values continuously exceeded the upper 90% percentile of the expected temperature values for more than five consecutive days. Correspondingly, a marine cold spell was defined when the measured values dropped below the lower 90% percentile for more than five consecutive days. Anomalous period calculations were also conducted for high-salinity phases and low-salinity phases via the same methodology.

Attached to the profiling system, the stereoscopic camera system was randomly positioned in one of the five depth strata at 1 m, 3 m, 5 m, 7 m and 9 m above ground for 24 h each day. The image pairs covered a water volume of approximately 4 m^3^ which was automatically scanned for fish (and all other macroscopic organisms). In this way, all five depth strata were sampled within one week with two reserve days (weekend) in case a depth stratum was missed due to technical failures of the system. Stereoscopic images were taken every 30 min and transferred to a server for further processing.

Object recognition was performed semi-automatically via neural network-based “user-centred design methods”^52^. The architecture allows recognition of an organism, on which the model is trained, by identifying the head and the tail of an organism as well as the respective mutual allocation to ensure that a specific head is assigned to the correct tail. Using this method, the correct xy-coordinates of an organism’s head and tail were automatically extracted from the image. In the next step, the organism identified in the left image was located in the right image using image rectification^53^ and epipolar coordinates^54^. This process enabled the determination of the target areas for the head and tail of the organism in the right image on the basis of their positions in the left image. The previously described object recognition algorithm was subsequently applied to extract the xy-coordinates of the head and tail of the same organism in the right image. This allowed the object’s size to be calculated using standard stereogrammetric algorithms^41^. For a comprehensive description of the methods mentioned, the relevant and detailed publications on image analysis and object recognition should be consulted. These mathematical methods and procedures are advancing at an extraordinary pace, particularly in the field of artificial intelligence, which has evolved into a global, stand-alone research focus with applications spanning almost all areas of daily life, including ecological research.

Using the above-described image analysis methods, the seven most abundant organism groups, appendicularia, chaetognatha, decapoda, pelagic crustacea, pisces, pteropoda and scyphozoa, were automatically distinguished with their respective size‒frequency distributions. Rarely observed organisms were assigned to the “others” group. This group included annelida, aves, gastropoda, salps, thaliaceae, polychaetes and a few other species that could not be clearly identified in the stereoscopic images. As none of the available methods for automatic object recognition on images or size measurements via stereogrammetric methods are free of errors, all automatically classified organisms were manually verified and corrected in cases of incorrect group membership or length measurement. In this verification process, all individuals were also determined at the species level or to the lowest taxonomic level possible.

Based on the above dataset (see the data availability section), the mean biotic abundance per week was calculated as the catch per unit effort (CPUE)^55^. The CPUE was defined as the sum of organisms of one species (or group, if species identification was not possible) observed on 336 image pairs per week (2 images hour^− 1^ * 24 h * 7 days = 336 images per week). One week was used as the smallest sampling unit because the camera system scanned all five depth strata of the water column for at least 24 h per week. If a depth stratum was sampled with fewer than 48 image pairs per day, e.g., due to system failures, the CPUE was recalculated to 336 image pairs per week.

Our studies at the same site^23 ^as well as the studies cited herein, showed that the annual cycle of polar biota does not follow the Christian calendar (1st of January to 31st of December) but is characterized by a polar cycle with permanent light during the polar summer and permanent night in the polar winter. Our previous studies^23^ clearly revealed that within this period, biotic abundances increased from the lowest values in August to the highest abundances and species diversity values during the polar winter and then strongly declined again in the spring. This has been described for most higher trophic level nonvertebrates, such as the sea-spider *Hyas araneus*, but especially for fish, such as *Gadus morhua.* For example, age 0 *Gadus morhua* migrates into the littoral zone around August/September of one year and leaves the littoral zone at age 1 no later than April–May of the following year. We therefore used the period beginning in the polar summer (1. August), lasting through the entire polar winter and ending in July (31. July) the next year and defined this as a “cohort year” when addressing biota. For example, the 2014 cohort year started on 01. August 2014 and ended on 31. July 2015.

### Calculating anomaly metrics for biota

To examine the possible effects of water temperature and salinity anomalies on the shallow-water community, numerical representatives of anomalies were also defined for the community. Following Hobday^10^ we defined a biotic anomaly when the measured abundance value within a week exceeded the upper 90% percentile value or dropped below the lower 90% percentile of the long-term average abundance value of the respective week calculated from 2012 to 2020. Because our system setup allowed a full stereo-optical assessment of the entire water column only within seven days and not within five days, we had to extend the Hobday 5-day phase for the calculation of abiotic anomalies to 7 days for our calculation of biotic anomalies.

### Analysing the interrelationships between abiotic and biotic anomalies

The effects of abiotic anomalies on a community are not necessarily instantaneous and may occur with an unknown temporal delay. Assuming a non-migratory population, a temporal delay would mean that anomalies might lead to an increase or decrease in total organism abundance across all species in the same area a few months or even a year later when, for example, the spawning success or survival of age-0 fish are affected. To address the problem of an unknown temporal offset between abiotic anomalies and biotic responses, we applied the concept of “day degrees”^56^ within one cohort year as an analytical tool. This concept recognizes that an environmental proxy does not necessarily cause an instantaneous physiological or behavioural response in a specimen. Instead, the cumulative exposure time of a specimen to the proxy, measured in ‘days per cohort year’, has a measurable effect. To compare the occurrence of anomalies in abiotic variables, temperature and salinity, with the occurrence of anomalies in biotic abundance within each cohort year, we summed the occurrence of anomalies (duration in days × intensity) per cohort year for both the abiotic stressors (sum of all temperature‒salinity anomalous values per cohort year) and the biotic abundances (sum of all abundance anomalies per cohort year). Because biotic anomalies are usually not normally distributed, as positive anomalies may reach high abundance values but negative values cannot fall below 0, we transformed both the sum values of the abiotic variables per cohort year and the sum variables of the biotic response variables prior to the statistical correlation analysis (Fig. 5). Even though data lose the intrinsic information on the numerical distance of two measurements along a continuous scale by rank transformation and therefore the statistical results usually have less power, rank transformation ensures the normal distribution of the datasets^57^ so that statistical correlation procedures, which are based on normally distributed data, are valid.

## Data Availability

All temperature and salinity raw data used in the manuscript to calculate anomalies are available in PANGAEA under the search term “Hydrographical time series data of the littoral zone of Kongsfjorden, Svalbard”. All biota data are available upon request by philipp.fischer@awi.de and have been submitted to PANGAEA for publication.
